# The impact of regulation, reimbursement, and research on the value of 3D printing and other 3D procedures in medicine

**DOI:** 10.1186/s41205-022-00132-0

**Published:** 2022-01-31

**Authors:** Frank J. Rybicki

**Affiliations:** University of Cincinnati, 2600 Clifton Ave, Cincinnati, OH 45221 United States

## Introduction

My opinions in this Editorial have been shaped by 20 years working in three academic Health Care Facilities (HCFs), with a clinical focus on medical image segmentation and image post-processing. During the first decade I studied and honed my skills in 3D visualization, defined as the ensemble of segmentation that can be viewed on a standard 2D display [1]. During the second decade I studied more sophisticated segmentation and medical image data refinement, including 3D printing [2] as well as virtual and augmented reality [3]. Over the past 5 years I have served as Editor in Chief of *3D Printing in Medicine*. I proposed and Springer/ BMC launched the journal with the intention of publishing peer-review original reports, review articles and guidelines, and intriguing case reports [4] that support our field that is growing both in size and diversity. This editorial presents my own opinion on several important developments and milestones since the journal launch, with the hope that my one perspective can inspire new contributions to the journal, and to the field in general.

## Regulation

While providers (physicians who work in HCFs) recognize that the FDA regulates medical devices, details regarding ‘how’ and even ‘why’ are not universally understood. While it has been 25 years since I graduated from medical school (as a radiologist there were 5 years of post-graduate training, hence the 20 years on Faculty in a HCF), I do not remember learning about the FDA as part of the curriculum, even in my own science-centric medical education program. In the United States (US), the FDA has regulatory authority to provide reasonable assurance of the safety and efficacy of medical devices. That authority is not limited to transactions between companies and HCFs. However, one often thinks of the role of the FDA is regulate devices that a company create, market, and sells either to the HCF (the hospital) or to the providers who use them [5]. The FDA regulates companies in the interest of safe healthcare for Americans, who in turn fund the administration. The FDA can and does require companies to provide evidence of efficacy for medical devices. While medical devices are broken into classes [6], the important concept for this editorial is “risk” – and by this I write as a HCF provider to mean the risk to the patient when the 3D service such as a 3D printed medical device is used in patient care.

Defining this risk for 3D printing in HCFs has been challenging. I strongly support the actions of the FDA to gather information on risk. I believe that the definitions of risk categories, and more importantly evidence regarding risk will drive how the FDA begins to regulate 3D printing in HCF. Although possibly oversimplified, for the purposes of this Editorial all 3D printing is considered to be patient-specific, and I have fabricated three lumped sets of procedures: “anatomic models”, based on the prose in the American Medical Association (AMA) Current Procedure Terminology (CPT™) codes 0559T and + 0560T [7], “anatomic guides” as defined in codes 0561T and + 0562T [7], and 3D printed implants that are coded with respect to body part.

Risk stratification and how it impacts value overall, medical HCF costs, and payments require a review of developments over at least the past 5 years. In 2016, after an important publication by the FDA in *3D Printing in Medicine* [8], I received permission (see Appendix 1, Guidelines – Point 9) from the Board of Directors of the Radiological Society of North American (RSNA) to co-Chair a joint meeting with the FDA to discuss 3D printing of anatomic models. The meeting was held at the FDA White Oak Campus in Silver Spring, MD on August 31, 2017. The meeting was open, and there were 30 members from the newly minted RSNA 3D Printing Special Interest Group (SIG) who attended, 24 of them in person (Appendix 2). One key strategy in creating the SIG included advocating for (and being granted) a new RSNA membership category so that people in medical 3D printing industry who otherwise could not previously become a member of the RSNA, could for the first time join the RSNA and participate equally with radiologists and imaging scientists. Other stakeholders at the August 31, 2017 meeting included industry employees who were not SIG members, and perhaps providers or other HCF representatives who were not members of the SIG.

The White Oak Campus joint meeting produced, in my opinion, two major outcomes. The first major outcome was that the FDA outlined the precursor of what will eventually evolve into a 3D Printing Medical Device Production System (MDPS). The MDPS is defined in the International Medical Device Regulators Forum [9], and it is a critical part of the 2021 Discussion Paper [10] that is open for public comment at the time of writing this Editorial (December 2021) and until early February, 2022.

The 3D printing MDPS is defined as, “a collection of the raw materials, software and digital files, main production equipment and post processing (if applicable) equipment intended to be used by a healthcare provider or healthcare facility, to produce a specific type of medical device at the point of care, for treating or diagnosing their patients, or preventing or mitigating disease, or to affect a structure or function of the body. An MDPS includes the medical device it is intended to produce.”

My interpretation is that a MDPS is a “toolkit with specific instructions” – see footnote 18 in [10], to be used by HCF employees to 3D print a medical device within the HCF (Table 1, Scenario 1). While written as a Discussion Paper [10], in my opinion the prose points towards the future and away from the present where the FDA has not published guidance substantially clarifying the agency’s regulatory oversight of 3D printing medical devices within HCFs.

**Table 1:** FDAs definition of three potential scenarios for 3D printing within HCFs [10]. Please note that the prose is copied directly from the Discussion Paper. However, footnotes from the FDA are not included. Please see the FDA website to download the full document

	Scenario 1: Healthcare facility using MDPS	Scenario 2: Traditional Manufacturer Co-location	Scenario 3: HCF assumes all manufacturer responsibilities
Entity designing/ developing the device	Traditional Manufacturer	Traditional Manufacturer	Healthcare Facility
Entity using the 3D printing system to produce devices	Healthcare Facility	Traditional Manufacturer, including any potential Contract Manufacturer	Healthcare Facility
Entity responsible for complying with applicable regulatory requirements	Traditional Manufacturer	Traditional Manufacturer, including any potential Contract Manufacturer	Healthcare Facility

My interpretation of Scenario 1 (Table 1) is that the HCF facility buys the MDPS from industry (e.g. a 3D printing company with a medical business vertical), like it buys other medical devices in a radiology department such as a vial of gadolinium, a fluoroscopy unit, a CT scanner, or a Picture Archiving and Communication System (PACS). The company or “traditional manufacturer” [10] completes the regulatory step of the MDPS listing/ clearance by the FDA. There are important factors for the HCF in this (for now, hypothetical) situation: what comes in the kit (software, hardware, materials/ resins, and instructions), and the cost of the kit.

The reason that I believe that the 2017 meeting laid the groundwork for the MDPS is that it was at this time the FDA introduced a new strategy, and what is the current strategy for companies to market and sell a "system" that a hospital can use for 3D printing anatomic models. The actual “device” that is cleared by the FDA is the software, but it is cleared for an intended use with a combination that included validation of a specific 3D printer, the material for 3D printing, and the specific anatomy. I feel strongly about image segmentation, having focused on it as a career pathway in academic medicine. I also feel strongly about the software used to perform these tasks, and I recognize that FDA cleared software should be used for all parts of medical image segmentation that impact patient care.

The RSNA SIG had two official meetings held at the Scottsdale Ranch Community Association (Scottsdale, AZ) that straddled the White Oak meeting. One was in March 2017; the second was in March 2018. At the March 2017 Scottsdale Ranch meeting, the SIG vetted and voted to recommend the use of FDA cleared software for 3D printing in HCFs. This recommendation was published in 2018 [11] and to me represents how healthcare professionals working within a medical society can take a positive step to impact patient safety.

The second major outcome from the August 2017 White Oak meeting was ‘how’ the system (software cleared, with the combination of the hardware and materials for an intended use on a specific anatomy) was cleared. The FDA announced that the system would be cleared using product code LLZ, “System, Image Processing, Radiological” [12]. This was very important to me as a radiologist because it represented a departure from an earlier, more foreign product code to one used in medical imaging. Produce code LLZ is used for 3D visualization software, and to me this meant that the FDA recognized that image segmentation and post-processing was one of, or even “the” key step in medical 3D printing. This point was vetted at length at the March 2017 Scottsdale Ranch meeting.

While I recognize that I am biased, I firmly believe that the medical image segmentation that form the basis of 3D procedures have been undervalued since they were launched in clinical radiology [13]. Diagnoses are made from volumetric medical images. To make a diagnosis, the observer (e.g. the radiologist) mentally segments organs and other anatomy from a (sliced) volume, and registers that mental segmentation against a fund of knowledge of what is normal or abnormal. This mental segmentation is what a radiologist does, and the training and practice is a big part of what separates a radiologist from other people who look at medical images. When appropriate, the extra step of digital segmentation (what we usually call “segmentation”) – and all the different ways that you can look at it, hold, it, or transform it into a guide or implant, is the best way to communicate how humans are impacted by disease and the best way to implement patient-specific treatment.

The digital segmentation, or segmentation, is the common thread for all 3D procedures. It is the most important step, and it introduces error. However, the majority of those errors are human errors because the person doing the segmentation lacks the correct medical training. This is the “at risk” step, although there is a paucity of research and other literature on this risk.

The FDA did not have a major release related to medical 3D printing in calendar year 2018, but in May 2019 there was a precursor concept presentation attributed to the FDA Additive Manufacturing Workgroup. This was not a formal FDA publication, but instead was concepts being preliminarily discussed within the workgroup. There were 6 lettered scenarios, lettered A – F. Scenario “F” was “Others”, leaving 5 descriptive scenarios (Table 2). On one hand, these lettered scenarios have been supplanted by the numbered 3 scenarios in the 2021 Discussion Paper [10]. On the other hand, my feelings are that parts of these scenarios are still important to consider.

**Table 2:** Summary of A-E from the Potential 3D Printing Scenarios proposed by the FDA in 2019

A: Minimal risk 3D printing at HCF	
B: Device designed by manufacturer using validated process with turn-key system	
C: Device designed by manufacturer using validated process that requires additional capabilities within the HCF	
D: Manufacturer is co-located at a HCF	
E: HCFP becomes a manufacturer	

To me it makes the most sense to approach risk and regulation from devices in a descending risk profile – while there are exceptions, the general risk profile is implants (highest risk), followed by anatomic guides, and finally anatomic models. Higher risk devices are largely produced by industry, although HCFs are now beginning to 3D print anatomic guides. I interpret the evolving regulation as focused on higher risk medical devices, although it is likely to impact all 3D printing in HCFs. Specifically, if providers in HCFs 3D printed only anatomic models, I believe that the “regulatory” section of this Editorial would be a lot shorter.

So why engage in HCF 3D printing at all? My answer is that when brilliant surgeons [14] get a mastery of technologies and develop a longstanding relationship with their consulting providers to include radiologists [15], there is very little that the surgeons can’t do [16]. Higher risk devices such as guides are also more expensive than anatomic models, although it remains to be seen if supplanting any or all 3D printing from a company with those 3D printed in a HCF will decrease the overall medical costs. Those data will be essential as HCFs report their 3D procedure experiences.

Since the 2017 White Oak meeting, I have been very confident that the FDA would further clarify oversight approaches for higher risk medical devices (guides and implants) printed in HCFs. While the 2017 White Oak meeting focused on anatomic models, my impression was that the terminology and groundwork laid at that meeting would extend as the more advanced hospitals were investing in equipment capable of producing anatomic guides. I believe that tertiary HCFs can 3D print advanced medical devices, and in some select centers there will be sufficient case load with high complexity so that the investment in time and money to assume all manufacturer responsibilities (Scenario 3, Table 1) may be suitable. The remaining, large majority of HCFs will use a MDPS.

This leaves anatomic models (e.g. those for surgical planning where there will be no guide and no implant). Here is my own anatomic model tip sheet: lowest (but non-zero) risk, lower in cost than guides and implants, current low technical and professional collections – based on new Category III billing codes [7], largest volume but smallest per-patient impact on industry if produced within a HCF, becoming ubiquitous as HCFs install more 3D printers, important for HCFs to expand creativity and enhance complex procedures, a growing set of established clinical indications (albeit with limited evidence based outcomes), and potentially very challenging to regulate by the FDA.

Even if you disagree with my tip sheet, please consider Scenario A in the Conceptional Framework (Table 2) that reads “Minimal risk 3D printing by Healthcare Facility Personnel”. When I read this prose from the FDA, I don’t get the gestalt that a patient specific anatomic model, even one with no modifications [5] to the anatomy from a CT scan, would fall into Category “A”. Should it? If the anatomic model segmented by a radiologist from a CT scan without modifications and 3D printed to be used in an office or radiology reading room only for surgical planning does not fall in Category “A”; then it must fall into another category. I believe that scenarios B and C, “Device designed by manufacturer using validated process” are also glimpses at the MDPS from the Discussion Paper [10]. Scenario C could then include a riskier or more complex future MDPS - because additional capabilities are required, for example sterilization and biocompatibility, among others. I believe that Scenario D and E from the 2019 Conceptual Framework are akin to Scenarios 2 and 3 in the Discission Paper [10], respectively. “Healthcare Facility Personnel becomes a manufacturer (Table 2)” or “HCF assumes all manufacturer responsibilities (Table 1)” as mentioned above seems daunting for a vast majority of HCFs, particularly one with providers who may be allocated only a small amount of protected time to expand a 3D visualization service line to include 3D printing and virtual reality.

Perhaps the most strategic approach could include select patient populations where the return on investment for a FDA-based regulation is lower (than a threshold that could be vetted) and more robust self-regulation is possible. Within radiology, there is a strong precedent for accreditation from the American College of Radiology [17]. There are also important and related training and certification considerations – for the purposes of brevity I am leaving them out of the scope of this Editorial but assuming that these needs will be met. One example would be an enhanced American Board of Radiology Diagnostic Medical Physics certification program as these diplomats already populate radiology departments.

This is where data and dialogue on risks is needed. I believe that if a HCF intends to only make anatomic models that are used in an office or in a radiology reading room, the expertise required should be tailored accordingly. In my experience, the factors that best mitigate this risk are a deep knowledge of the anatomy in health and disease states combined with an understanding of the pathophysiology of the disease and therefore how it commonly (and less commonly) appears on CT and MRI studies. These are the factors that enable 3D visualization and 3D printing of anatomic models to best contribute to health care. These considerations require literature support and would make a very important contribution to helping determine the value of 3D procedures.

## Value

“Regulatory & Reimbursement” are often paired. Although they are related, I don’t think about it this way. Instead, I focus this section on health care value as defined as (“Quality” / “Cost”), with that ratio multiplied by “Appropriateness”. From an overall healthcare perspective, Appropriateness as a multiplier has a range from 0 (an entirely inappropriate procedure has no value) to 1 (appropriate procedures deliver full value). For 3D services, I believe that “Quality” has 3 inputs: Safety, Usefulness or Utility (for the provider), and Benefit (for the patient).

For medical 3D printing, the reason that the work from the FDA is so important to me is that the FDA is taking necessary steps to benchmark the “Safety” component of the “Quality” numerator in the Value equation. While utility and benefit are still difficult to estimate, the value numerator becomes even more difficult when one can question the quality of 3D printed parts. I do believe that senior providers working in HCFs, the FDA, and other stakeholders will eventually secure medical device safety, i.e. taking it more ‘out of play’ within Quality. This can’t happen too soon, but how it happens is critical, especially because Value includes “Cost” and “Appropriateness”. It is important to discuss and gather data regarding how future 3D services may be provided. For example, there may be clinical scenarios for 3D printing of very low risk medical devices, such as some anatomic models printed by HCFs as a secondary representation of medical device data, that if used by the same HCF should not be actively regulated by the FDA (i.e. the agency uses enforcement discretion). Such anatomic models are not intended to provide primary diagnosis nor used to make treatment decisions. These models, in theory provide secondary, physical representations of patient-specific medical device data to supplement care team discussions.

Since the 2017 White Oak meeting, I have on several occasion heard the term “levels the playing field” between industry and HCFs, in the context of costs and effort to meet FDA guidance. While this represents one downstream implication, the reason that I support the recent Discussion Paper and future, carefully determined regulation is that it enables Value to be better defined and quantified in those patients for whom I have been able care for over the past 20 years. Assuming that safety is secured for 3D printing and other procedures, I propose that the numerator of the Value equation could be recast as “utility-benefit”.

I will try to define a utility-benefit metric that can better define quality, assuming that the 3D service meets safety criteria. Before doing so, allow me to harmonize a potential disconnect between two different ways to view the same thing, from the medical device perspective and from the patient care perspective. From the manufacturing side, one may consider an individual case in terms of the medical device. However, from the provider (HCF) side, the patient is defined by the diagnosis (clinical scenario) and a treatment plan. For the purposes of this discussion, one can assume that all patients will have an advanced imaging study (CT and possibly MRI). For example, consider a patient with tinnitus who is diagnosed with vestibular schwannoma with an MRI, after which a CT is also performed to map the skull (bone) for surgical planning. For this hypothetical patient, because of the large tumor volume and relationship with the venous drainage at the skull base, 3D procedures in addition to the traditional axial, coronal and sagittal MR and CT images are sought from the radiologist. The surgeon requests “3D Interpretation and Reporting of Imaging Studies” and if performed by the radiologist would be billed as CPT 76377 as a secondary code. For this clinical scenario, the segmentation and volume rendering may take one hour (or more) of the radiologist’s time, particularly if there is a need to fuse the CT (to best see the base of the skull) and a contrast-enhanced MRI (to best see the full extent of the tumor and the draining veins).

I follow a surgical planning format (Table 3) for every patient in whom 3D procedures (to include 3D visualization and 3D printing) is considered. It follows the patient experience and how payors assess these (billable) procedures. While anatomic guides and patient-specific implants are physical objects and often use 3D printed to create them, is it necessary and appropriate to always 3D print an anatomic model? My experience, including working with limited time and limited physical resources, suggests that 3D printing may not always be needed when it is being performed for anatomic models. After a CT or MRI, there is request by the referring clinician for additional image segmentation. This procedure can be performed by the radiologist using current CPT codes 73676 and 73677. (This assumes that the CT and MRI were not performed and primarily coded as angiographic studies. For CT and MR angiography, the additional work is baked into the primary diagnosis code. For example, when billing 75574 “CT Angiography Cardiac – TAVR” it would be incorrect to add a secondary code 76377 after the radiologist segments the annulus of the aorta.)

**Table 3:** Symptomatic Hearing Loss and Vertigo – Differential Diagnosis includes Vestibular Schwannoma. Surgical Planning for a Hypothetical Patient includes MRI and CT

Procedure	Appropriateness	Billing code(s)
MRI with and without contrast	Usually appropriate [18]*	70553
CT with and without contrast	May be appropriate [18]*#	70482
3D visualization	NA (secondary code)	73677&
Virtual reality	TBD	None
3D printed anatomic model	TBD	0559T + 0560T

*Refers to the appropriateness rating in the American College of Radiology (ACR) Appropriate Use Criteria (AUC) where the MRI is rated “Usually appropriate” and CT is rated “May be appropriate”. # ACR AUC only considers initial imaging evaluation, and thus the CT scan is scored "May be appropriate". Because of the exponential complexity, the ACR does not nest AUC to provide recommendations after the initial imaging is complete. However, given the diagnosis of vestibular schwannoma from the MRI, the CT to better assess the skull base is routinely performed and I expect it would be reimbursed. & The secondary code 73677 may be added to the MRI or the CT scan. However, if either is performed an an angiography, 73677 could not be added.

I mentioned earlier that the work a) described by 76376/ 76377 and b) needed to have a meaningful consultation between the diagnostic radiologist and the interventionalist is often undervalued. Another way to look at these procedure codes is that they are performed for “tough cases”. If the case were not challenging and did not require extra work, that consultation would not be considered. Instead, the interventionalist (usually a surgeon) would review the images (along with the report) and operate on the patient, as is the case for over 99% of procedures that require medical imaging. On one hand, “tough cases” are part of the spectrum of all cases, and workload and collections are averaged over a larger experience (e.g. the work week of radiologists, all the radiology codes submitted over a time period). The issue for 3D visualization (what is done today within the scope of 76376/76377 and beyond to include virtual reality and 3D printing) is that most, if not all the cases are tough, image segmentation is time consuming, and consultation between the surgeon and the radiologist is needed, along with the report for the medical record.

As noted above, the common thread for all these procedures is image segmentation. Each downward procedure in Table 3 includes differing and increasing digital manipulation of the data, but the common denominator is the identification of the anatomy and segmenting pathology that is required for 3D visualization. Additional steps are needed to generate a suitable new file format (e.g.. STL), that in turn could be used to generate a 3D PDF that can be easily manipulated by the surgeon, or that can be viewed in virtual reality and further digitally manipulated. For some clinical scenarios, these procedures will still prove insufficient, and 3D printed anatomic models are needed for surgical planning. This comes with the additional expense of creating the physical part. The utility-benefit metric represents the numerator of the stepwise incremental value (top to bottom, Table 3) of procedures for intervention planning.

More useful tools (e.g. the anatomic model, or an anatomic guide) will lead to a higher Value numerator, and in addition to the being useful, the service will have to provide a benefit over those steps that require less work and fewer resources. Each increment also has an incremental cost. Although not applicable for this clinical scenario, if an anatomic guide were needed to access the tumor or perform reconstruction, a 3D printed anatomic guide could be added to the bottom of Table 3. Research and guideline (consensus) documents should focus on this new numerator (utility-benefit), the costs, and the appropriateness for the incremental set of pre-procedure procedures where advanced visualization and 3D printing may be used. Focusing on these three metrics, and those factors that influence the metrics, is the best - and maybe the only, way to achieve fair reimbursement. Both the Quality and the Cost of 3D printing (in a HCF) will be refined after regulation is clarified by the FDA, and the net result should be add value.

More open discussion on costs will also be beneficial, even with the recognition that industry prices are not shared. Hospital pricing is become more transparent, although with some resistance [19]. HCFs consider costs and revenue parsed as professional (those borne / collected by a physicans’ association or practice plan) and technical (those usually borne/ collected by the hospital). For 3D printing in HCFs, both professional and technical costs typically begin very small (e.g. one provider with a desktop printer) and then expand to include engineering/ technical support as well as more expensive equipment. Provider resources are limited, and initial funding within a HCF may include very little time away from a clinical service when compared to the time needed for the additional segmentation and 3D printing.

The Appendix of the Discussion Paper [10] breaks down 3D printing into 5 stages; however, translating these to professional versus technical work has challenges. For example, if the surgeon requests 3D printing from a radiology-based service, the professional work and cost includes the clinical consultation before and after the device is printed, and includes a substantial amount of the segmentation. This would typically include part of FDA #1 (device design stage), part of FDA #2 (software workflow stage), and part of FDA #5 (process validation and acceptance activities stage). It is important to study and tackle the separation of professional versus technical work early on, as costs will vary greatly according to how the work is divided between a radiologist and an engineer or technologist. The technical cost related to software, hardware, and materials can have even greater variation. The complexity of the medical device must be considered, and there is an unmet need to expand the literature with respect to specific clinical scenarios and the appropriateness of the service.

Regulation and reimbursement have a point of intersection with respect to “diagnostic use”. At the August 2017 White Oak meeting - near the time when the FDA announced that 3D printing migrated to produce code LLZ, there was a substantial discussion on what constituted Diagnostic Use for 3D printed anatomic models. The definition included a) diagnosis, b) patient management, and c) patient treatment. I interpret this to be all parts of what a HCF terms “patient care”, with the possible exception of using the patient-specific model as a tool for educating the patient and obtaining consent. I believe that the FDA clarified their definition of diagnostic use for 3D printed anatomic models to make it clear that all patient specific 3D printed parts (to include anatomic models for which the image-based anatomy is not altered) fit into this wide definition of diagnostic use. Thus, the patient-specific 3D printed parts would all be considered medical devices and would therefore fall under the purview of the FDA.

For the very large majority of patients for whom 3D printing is appropriate, the diagnosis is secure and is not altered by the activities of 3D printing. In my experience, there are very unusual cases for which I have begun an image segmentation after reading a radiology or cardiology imaging report, and upon review of the images and the report I have asked that a diagnosis be modified or an additional imaging study be performed. On the other hand, if 3D printing is not used for patient management, patient treatment, or both, the procedure is not appropriate and has little to no value. Collections are very unlikely for procedures without evidence basis for value.

Medical 3D printing that is appropriate (i.e. used for patient management and patient treatment) is considered “diagnostic use” and the parts are medical devices. When made by industry, they are regulated by the FDA. Quoting the part of the Discussion Paper [10] that in my opinion has the most ‘teeth’, “FDA regulation is designed to provide a reasonable assurance that devices are safe and effective; This assurance applies regardless of where and how a product is manufactured.”

As I mentioned earlier, securing medical device safety as one variable within “Quality” can’t happen too soon, but how it happens is critical. Early, open discussion will enable better reimbursement planning. A second point of intersection between regulatory and reimbursement is that FDA approval is often required for new CPT codes, and I anticipate that approvals and costs for MDPSs will impact category I AMA CPT™ billing code discussions. Anticipating how the costs may change for all three scenarios (Table 1) is essential for HCFs to plan and budget 3D service lines.

HCFs may pay more money for the same medical device under a “live” Scenario 1 (HCF using MDPS) because of the future cost of the MDPS. This will depend on what constitutes the final products for sale and the pricing competition among companies. It is reasonable to consider that for HCFs, the cost of 3D printing may increase, with the money “spent” on patient safety, specifically removing uncertainty of a faulty 3D printed part from the numerator of the Value equation. To maintain Value, the overall quality improvement should match an increase in cost. Cast another way, planning and research should consider the lowest reasonable cost to ensure safety (and at least maintain quality, if not improve it) with respect to the clinical scenario for which 3D procedures are appropriate. Costs themselves could then be tiered and then vetted for reimbursement from Category I billing codes for 3D printing.

As noted earlier, in a “live” Scenario 3 (HCF assumes all manufacturer responsibilities), the HCF cost may be well outside of a hospital budget, especially for HCFs that have less experience and pecuniary constraint. As noted earlier, graded responsibilities within a HCF could be considered.

## Research

High level research is required for successful and effective Category I billing code applications. Research that includes clinical outcomes & utility metrics (both of which provide evidence-based appropriateness), and costs should be prioritized. While there continues to be an increase in the number of publications that study 3D procedures, in my opinion many of these papers solidify clinical scenarios but do not necessarily define and quantifying utility and outcomes. For example, cardiovascular applications are being crystalized in the literature as more detailed segmentation and its representations are showing clinical benefits. However, there is still a need for data-driven appropriateness, and this should include considerations of the technical needs for each clinical indication.

Two general criteria for category I CPT codes are a demonstration that a large number of providers/ HCFs performs the service, and high-level research on Value, preferably to include as many metrics as possible to estimate value. One approach to bridge this gap is the joint ACR-RSNA 3D Printing Registry [20]. Registry data should show broad usage from as many HCFs as possible. Since the registry is maintained by the American College of Radiology, this may require radiologists to partner with subspecialists with a unified goal of expanding the knowledge base, and in particular to demonstrate widespread use of medical 3D printing.

A paucity of multicenter trials that demonstrate utility, cost, and outcomes for 3D services would negatively impact reimbursement. While I don’t fully understand the barriers, I will share some observations. In my experience, several initial multi-center data sets and early, important studies that can drive reimbursement are driven by and entirely funded by industry. In some studies, the funding company desires to gain market share from their competitors for sales of their medical device (e.g. a CT scanner). For other studies, with recognition of a desired budget neutrality for United States healthcare, a company can fund a trial that provides an alternative diagnostic or treatment strategy – with the intent to shift care pathways (procedure codes) away from one technology (or competitor company) and towards a technology sold by the funding company.

To my knowledge, neither strategy has been realized for 3D services, including 3D printing. This is not at all a criticism directed towards industry; in fact all of the seed funds for the registry [19] were donated by 3D printing companies. The reality is that clinical trials are expensive, and without clear market benefits they are difficult to justify to company shareholders. That clarity may in part by clouded by the evolving regulatory considerations. I could imagine that more than one ‘live’ MDPS will be available in a future Scenario 1 [10]. At this point, and perhaps with Category I billing codes on a shorter horizon, industry may be more eager to fund trial. Part of the issue may also be education. Lastly, while I do not have an supporting evidence, my suspicion is that a majority of company revenue from most, potential industry sponsors is driven by 3D printed guides and implants. Since these devices are already within the company portfolio, an industry-organized trial that could potentially move the services to a HCFs may be considered undesirable by those companies with an interest in study outcomes.

If this is true, it leaves the burden on HCF investigators to organize smaller multicenter trials to harness preliminary data to apply for larger funding portfolios. This is a tough game to win, especially since academic time among HCF providers and scientists to write a grant is vanishing scarce. Because the qualifications for grant writers will include experience in clinical trial design, technical experience in medical devices, and a deep understanding of the clinical impact of 3D services, a shared approach may be needed. The RSNA SIG recently launched a small grant program – this program is very highly valuable, and much larger seed funds must be invested to secure initial resources that can then grow into larger trials.

## Summary

This Editorial provides my own personal opinions and is written to stimulate discussion and important research that will enhance the literature for 3D printing procedures. After a very brief review of the most important points in the changing regulation of 3D printing in HCFs, I attempted to connect the dots between maintaining safety and efficacy and the larger value equation for 3D procedures. The common thread in these procedures is digital segmentation of patient anatomy captured in volumetric medical imaging; properly assigning the work and clinical impact of segmentation is a key part of fair reimbursement. Another part includes studying and publishing literature focused on all metrics that impact 3D procedure value. This research must show widespread use and enhanced quality of care when 3D procedures are used in addition to the source imaging itself. Recognizing that my personal opinion is one of many, I separate 3D Procedure Quality (the numerator of the 3D procedure Value equation) into 3 parts: Safety of the medical device, Usefulness or Utility for the provider, and Benefit for the patient. By ensuring safety, Value can be reduced to utility and benefit, that can be considered as its own metric for Quality. I believe that the most rapid progress will reply on maximizing this numerator while minimizing Costs.

## Supplementary Information

**Additional file 1.**

## References

[1] Giannopoulos AA, Chepelev L, Sheikh A (2015). 3D printed ventricular septal defect patch: a primer for the 2015 Radiological Society of North America (RSNA) hands-on course in 3D printing. 3D Print Med.

[2] Mitsouras D, Liacouras P, Imanzadeh A, Giannopoulos AA, Cai T, Kumamaru KK, George E, Wake N, Caterson EJ, Pomahac B, Ho VB, Grant GT, Rybicki FJ (2015). Medical 3D printing for the radiologist. Radiographics..

[3] Sutherland J, Belec J, Sheikh A, Chepelev L, Althobaity W, Chow BJW, Mitsouras D, Christensen A, Rybicki FJ, La Russa DJ (2019). Applying modern virtual and augmented reality technologies to medical images and models. J Digit Imaging.

[4] Rybicki FJ. 3D Printing in Medicine: an introductory message from the Editor-in-Chief. *3*D Print Med. 2015;1:1. 10.1186/s41205-015-0001-5 PMC6036611 30050970

[5] Christensen A, Rybicki FJ (2017). Maintaining safety and efficacy for 3D printing in medicine. 3D Print Med.

[6] The United States Food and Drug Administration, “Classify your medical device”: Website: https://www.fda.gov/medical-devices/overview-device-regulation/classify-your-medical-device. Accessed 24 Dec 2021.

[7] Mitsouras D, Liacouras PC, Wake N, Rybicki FJ (2020). *RadioGraphics* update: medical 3D printing for the radiologist. Radiographics..

[8] Di Prima M, Coburn J, Hwang D, Kelly J, Khairuzzaman A, Ricles L (2015). Additively manufactured medical products–the FDA perspective. 3D Print Med.

[9] International Medical Device Regulators Forum. “Personalized Medical Devices - Regulatory Pathways” IMDRF/PMD WG/N58FINAL:2020. Website: https://www.imdrf.org/sites/default/files/docs/imdrf/final/technical/imdrf-tech-200318-pmd-rp-n58.pdf. Accessed 8 Jan 2022.

[10] United States Food and Drug Administration, “Discussion Paper: 3D Printing Medical Devices at the Point of Care” Website: https://www.fda.gov/medical-devices/3d-printing-medical-devices/3d-printing-medical-devices-point-care-discussion-paper. Accessed 24 Dec 2021.

[11] Chepelev L, Wake N, Ryan J, Althobaity W, Gupta A, Arribas E, et al. RSNA Special Interest Group for 3D Printing. Radiological Society of North America (RSNA) 3D printing Special Interest Group (SIG): guidelines for medical 3D printing and appropriateness for clinical scenarios. 3D Print Med. 2018;4:11. 10.1186/s41205-018-0030-y PMC6251945 30649688

[12] Food US, Administration D. Product classification – system, image processing. Radiological Website. https://www.accessdata.fda.gov/scripts/cdrh/cfdocs/cfPCD/classification.cfm?ID=LLZ. Accessed 24 Dec 2021.

[13] Fishman EK, Magid D, Ney DR, Chaney EL, Pizer SM, Rosenman JG, et al. Three-dimensional imaging. Radiology. 1991 Nov;181(2):321–37. 10.1148/radiology.181.2.1789832. 10.1148/radiology.181.2.1789832 1789832

[14] Thantchaleishvili V, Rajab TK, Shekar PS (2017). Lawrence H. Cohn, MD—our mentor in cardiac surgery. Ann Cardiothorac Surg [Online].

[15] Rybicki FJ (2018). Medical 3D-printing and the physician-artist. Lancet..

[16] Pomahac B, Pribaz J, Eriksson E, Bueno EM, Diaz-Siso JR, Rybicki FJ, et al. Three patients with full facial transplantation. N Engl J Med. 2012;366(8):715–22. 10.1056/NEJMoa1111432. 10.1056/NEJMoa1111432 22204672

[17] American College of Radiology Practice Parameters and Technical Standards. Wesite: https://www.acr.org/Clinical-Resources/Practice-Parameters-and-Technical-Standards. Accessed 29 Dec 2021.

[18] Expert Panel on Neurologic Imaging, Sharma A, CFE K, Aulino JM, Chakraborty S, Choudhri AF, et al. ACR Appropriateness Criteria® Hearing Loss and/or Vertigo. J Am Coll Radiol. 2018;15(11S):S321–31 9.916/j.jacr.2018.09.020. PMID: 30392601. 10.1016/j.jacr.2018.09.020 30392601

[19] Nikpay S, Golberstein E, Neprash HT, Carroll C, Abraham JM. Taking the Pulse of Hospitals' Response to the New Price Transparency Rule. Med Care Res Rev. 2021; 977558721924786. 9.1177/977558721924786. Epub ahead of print. PMID: 34148382. 10.1177/10775587211024786 34148382

[20] American College of Radiology – Radiological Society of North America 3D Printing Registry. Website https://www.acr.org/Practice-Management-Quality-Informatics/Registries/3D-Printing-Registry. Accessed 29 Dec 2021.

