# Supplementary material for: The impact of regulation, reimbursement, and research on the value of 3D printing and other 3D procedures in medicine
Source: 3D Print Med. 2022 Jan 31;8:6. doi: 10.1186/s41205-022-00132-0 (PMC8805272; doi:10.1186/s41205-022-00132-0)
Supplement: Supplementary file 1 — Additional file 1. [file 41205_2022_132_MOESM1_ESM.docx]

**Appendix 1**

Guidelines for Special Interest Group (SIG) on 3D Printing

Frank J. Rybicki, Special Interest Group Founding Chair

November 26, 2016

RSNA will establish a SIG on 3D Printing, so long as a petition is received stating the purpose of the SIG with an expression of interest from 25 RSNA members who will join. SIG members will pay dues and operate within the structure and guidelines approved by the Board of Directors. The 3D Printing SIG will serve as a three year pilot program to determine the viability of RSNA SIGs as a mechanism for enhanced member engagement and to guide establishment of overall criteria for SIG programs.

1. RSNA membership is required in order to be eligible for membership in the SIG. Annual dues are set at $40, of which $30 will be allocated to the SIG for its use.
2. The SIG will have a governing committee of at least three members, including a Chairman and Secretary. The SIG will elect its own leadership, determine the form and extent of elections to rejuvenate its leadership in a manner satisfactory to the Board, and define its proposed methods of governance in other respects.
3. The SIG shall each year propose a program of activities for approval by the Board of Directors.
4. An information page on RSNA.org will be provided with basic information about the SIG and link to a SIG members-only communication platform.
5. A platform for communication among SIG members will be provided by RSNA, which will include mechanisms to facilitate sharing of documents, announcements and any newsletter developed by SIG members.
6. At the RSNA Annual Meeting, the SIG will be provided with a two-hour time slot to conduct an annual business meeting, and any education programs organized by the SIG for the group. These will be listed in the Annual Meeting program. No CME will be granted for a SIG session.
7. Any other programming or publications intended for an audience broader than the SIG membership may be proposed through established RSNA processes.
8. The SIG may propose an educational program or workshop to be conducted between annual meetings, which meets the requirements of SACME and can could be captured as a webinar or online module open to all RSNA members. Program proposals must include a statement of staff’s role and the services to be provided in implementing the program, and be approved by the Board of Directors.
9. Corporate funds available as a result of firm commitments obtained prior to the creation of the 3D Printing SIG may be accepted for the use of the SIG. Any additional solicitation of outside funds can be done only to support an approved educational program or workshop, with prior approval of the Board of Directors and in coordination with RSNA’s overall corporate sponsorship efforts.
10. SIG members may not permit themselves to be identified as speaking on behalf of RSNA if they are participating in advocacy activities or making public statements, unless they have been authorized to do so by the Board of Directors.
11. Staff support will be provided by the RSNA Executive Department. It will be limited to necessary coordination of services outlined above. Agendas, minutes and reports will be the responsibility of SIG members.
12. The RSNA President-Elect/Secretary-Treasurer will serve as the SIG liaison to the Board of Directors.

RSNA 3D Printing Special Interest Group (SIG)

Rules of Operation

Frank J. Rybicki MD, PhD, Founder

**MISSION STATEMENT**

To promote the highest quality 3D printing applied to medicine via education, collaboration, and research. The SIG will focus on maintaining a prominent role for radiologists in this diverse and growing specialty. The group will also seek to provide physicians and allied health scientists with optimized educational and research programs.

**Method of Governance**

The SIG will be governed by a four member Executive Committee consisting of the following positions, each serving a one year term.

Chairman

Vice Chair

Secretary

Treasurer

The Steering Committee will be comprised of the 4 members of the Executive Committee plus the Chairs of the following 3 committees who will serve as voting *ex-officio* members:

Guidelines Committee

Industry Relations Committee

Trainee Committee

**Elections**

Each year during the RSNA Annual Meeting, the 3D Printing SIG will hold an Annual Business Meeting. A quorum will consist of the members present and voting. During this meeting the new Treasurer will be nominated by the Executive Committee and elected by the members present and voting. Other nominations may be accepted from SIG members and must be submitted to the Secretary no less than 10 days in advance of a scheduled business meeting. At the conclusion of the RSNA annual meeting the Vice Chair will become Chairman, the Secretary will become Vice Chair, and the Treasurer will become Secretary.

The Chairs of all committees will be appointed by the Executive Committee.

**Membership**

RSNA membership is required for all SIG members. There is a $40 annual SIG membership fee for all RSNA membership categories. RSNA will create a new Membership Category for individuals who do not otherwise fit into a current category. The dues is equal to the full membership, plus the $40 SIG dues. The new membership includes free registration to the annual meeting plus all benefits related to the SIG.

**Standards**

The SIG will work to establish a standard nomenclature/lexicon for 3D printing, based on current literature usage as well as industry standards that can be considered for RSNA adoption. The SIG will work to establish quality standards for medical 3D printing.

**Newsletter**

The SIG will electronically publish two newsletters per year, informing members of upcoming 3D printing venues and to keep the membership informed of new technologies.

**RSNA 3D Printing Special Interest Group (SIG)**

**Business Meeting Agenda**

**Sunday, November 27, 2016**

**1:00 – 3:00 pm**

**Room S104B**

**McCormick Place, Chicago**

1:00–1:15 pm    Welcome, Mission Statement and Rules of Operation

*Frank J. Rybicki, MD, PhD*

*Chairman, Special Interest Group in 3D Printing*

1. Call to Order - Establish a Quorum
2. ACTION ITEM - Governance structure
3. ACTION ITEM - Slate of candidates

1:15-1:25 pm    Historical perspective

*Andy Christensen*

*Former President, Medical Modeling Inc.*

1:25–1:35 pm Overview of RSNA programming in 3D printing

*Bill Weadock, MD*

*Treasurer, Special Interest Group in 3D Printing*

1:35-1:55 pm   Identification of Focus Areas of Interest within 3D printing

*Jonathan Morris, MD*

*Vice Chair, Special Interest Group in 3D Printing*

1:55-2:10 pm    Educational meetings

*Jane Matsumoto, MD*

*Secretary, Special Interest Group in 3D Printing*

2:10-2:25 pm    SIG for the non-radiologist (outreach)

*Gerald T. Grant, DMD, MS, FACP*

*Chair, School of Dentistry, University of Louisville*

2:25-2:45 pm    3D printing vendors and SIG interactions

*Fried Vancrean*

*Founder and CEO*

*Materialise*

*Scott Crump*

*Chief Innovation Officer*

*Co-Founder & Board Member*

*Stratasys*

2:45-3:00 pm   Summary, business meetings, future directions

*Jonathan Morris, MD*

**Appendix 2**


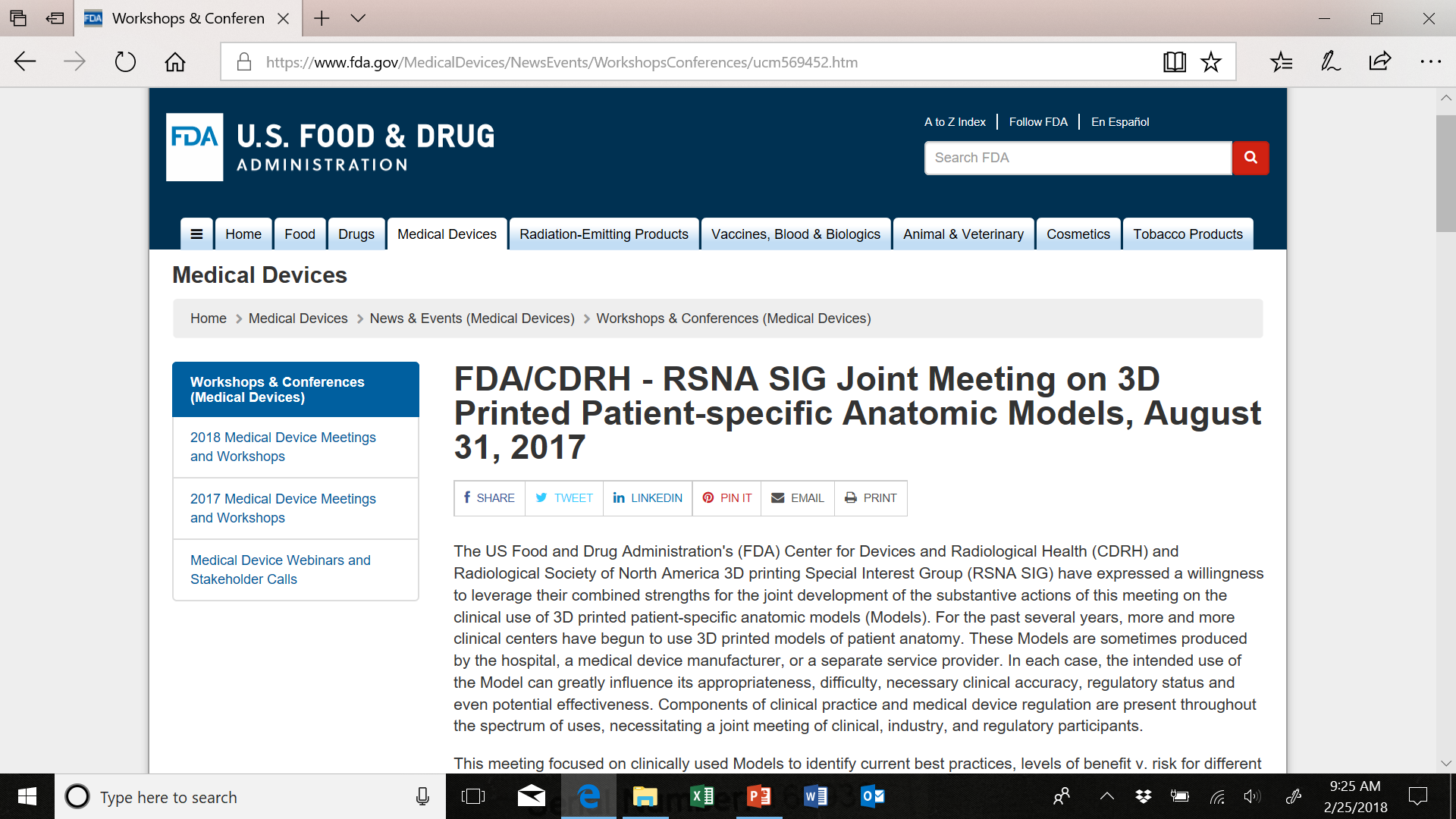


RSNA SIG MEMBERS who attended the FDA meeting on August 31, 2017

PHONE Althobaity, Waleed

Arribas, Elsa

Chanin, Brent

PHONE Chen, Hui (Jenny)

Christensen, Andy

Espinoza, Alejandro

Gaisford, Michael

PHONE Gillies, Melanie

Hirsch, Jeffrey

PHONE Johnnie, Joseph

Kircos, Louis

PHONE Kopacin, Vjekoslav

PHONE Letrong, Chris

Liacouras, Peter

MacCutcheon, David

Madhuripan, Nikhil

Matsumoto, Jane

McDaniel, Lauralyn

Mitsouras, Dimitris

Pietila, Todd

Rader, Scott

Ryan, Justin

Rybicki, Frank

Santiago, Lumarie

Sheikh, Adnan

Torluemke, Kimberly

Wake, Nicole

Wang, Kenneth

Weadock, William

Weimer, Katherine

White, Carissa

PHONE Wilke, Christopher
